# Bleeding Risk in Patients with Peripheral Arterial Disease

**DOI:** 10.3390/life13010047

**Published:** 2022-12-23

**Authors:** Adriana Visonà, Chiara Zurlo, Chiara Panzavolta, Annachiara Gobbo, Beniamino Zalunardo

**Affiliations:** Angiology Unit, Azienda ULSS 2 Marca Trevigiana, 31033 Castelfranco Veneto, TV, Italy

**Keywords:** bleeding risk, peripheral arterial, score

## Abstract

Patients with peripheral arterial disease (PAD) are at high risk of major adverse cardiac events (MACE) and major adverse limb events (MALE). Recently, antithrombotic therapies employing antiplatelet and anticoagulant drugs have proven to be valid in reducing MACE in patients with PAD and polyvascular disease and MALE, particularly in patients who have already been revascularized and remain at increased risk of MALE. However, more aggressive antithrombotic therapies lead to an increased risk of bleeding. Antithrombotic therapy and revascularization procedures entail an increased hemorrhagic risk that is also linked to having received more vigorous antithrombotic therapies. Therefore, it appears crucial to have specifically targeted scores for a PAD patient to assess bleeding and thrombotic risks. The correct utilization of a risk score will determine the variable risk factors for bleeding that can be corrected or modified, as well as identify patients at high risk that require regular reexamination and follow-up. Clinical risk scores do not represent the absolute reality, and inter-score variability must be taken into account. Moreover, several risk scores have been created to be basic and to facilitate and improve clinical decisions in daily practice. Many risk scores based on points vary according to the configuration of the studies, population type, and ethnic group, and many of the risk factor elements in a specific score are unlikely to sustain same weight for that risk. The best approach continues to be devising an uncomplicated, functional, validated, and precise score that can be adjusted to different clinical contexts and populations, while considering the mutable composition of clinical risk.

## 1. Introduction

**Figure 1:**
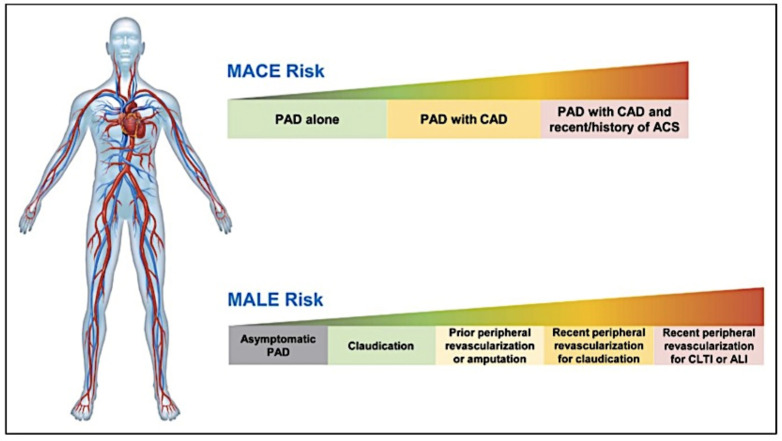
Ischemic risk in peripheral arterial disease (PAD) (reprinted with permission from Ref. [3]. Copyright 14 December 2022. Wolters Kluwer Health, Inc.)

Obstructive atherosclerotic arteriopathy of the lower limbs refers to atherosclerosis that leads to obstruction of the arteries of the lower limbs. This disease is commonly called peripheral artery disease (PAD). PAD has epidemic characteristics, and it can rightly be called an orphan epidemic since it is still underdiagnosed and undertreated. Recent data in the literature have shown that PAD affects an estimated 240 million people worldwide. The clinical presentation of the disease is variable and includes the typical symptoms of PAD, such as muscular pains that occur with exercise (called claudicatio intermittens) and atypical pains sometimes related to osteoarticular disorders that frequently occur at the same time, and symptoms typical of more severe forms of the disease, such as ischemic pain at rest or ulceration and gangrene with minor or major tissue loss, which are compatible with critical limb ischemic pictures. More severe forms of PAD can lead to amputation and acute limb ischemia, caused by rupture of the native atherosclerotic plaque and thrombus formation, or in situ thrombosis of stents or grafts in revascularized patients [1,2]. Although PAD shares the predisposing terrain of atherosclerosis with coronary artery disease and cerebral ischemia, there is growing evidence that PAD represents a peculiar connotation of atherosclerotic disease characterized by a high risk of limb-related complications (critical limb ischemia, acute limb ischemia, revascularization, and amputation, or MALE) and cardiovascular adverse events (myocardial infarction, stroke, and cardiovascular death, or MACE). In fact, the risk profile of these patients is heterogeneous. They often have coexistent coronary or cerebrovascular disease related to as polyvascular disease and are at increased risk of MACE. Revascularization improves symptoms in most patients suffering from PAD. In particular, peripheral revascularization improves the symptoms of claudication intermittens and the quality of life in patients with claudication intermittens, reduces ischemic pain, saves the limb in acute limb ischemia and chronic critical limb ischemia in many cases, and accelerates the healing of ischemic skin lesions in patients with critical ischemia. However, it does not prevent many patients from subsequently developing vascular complications of the limbs. Therefore, these patients are at heightened risk of MALE and acute limb ischemia, which is expected to occur four times more frequently in these patients than in subjects who have not undergone revascularization [3] (Figure 1).

When combined with the vigorous control of risk factors, particularly LDL cholesterol levels, high blood pressure values, glycemic control in diabetes, and the introduction of therapeutic lifestyle changes such as smoking cessation and body weight control, antithrombotic therapy plays a pivotal role in reducing MALE and MACE.

In recent years, the results of the COMPASS and VOYAGER studies have shown that more intensive antithrombotic strategies combining antiplatelet and anticoagulant therapies—the double pathway inhibition (DPI)—reduce MACE and MALE in selected patients [4,5] with polyvascular disease who have undergone recent peripheral revascularization performed primarily for critical or acute ischemia. In the COMPASS study, a primary outcome event of cardiovascular death, stroke, or myocardial infarction occurred in 379 patients (4.1%) who were enrolled for rivaroxaban plus aspirin treatment and 496 (5.4%) who were enrolled for aspirin treatment alone (HR: 0.76; 95% CI: 0.66 to 0.86; *p* < 0.001). There were 313 deaths (3.4%) in the rivaroxaban-plus-aspirin group compared to 378 (4.1%) in the aspirin-alone group (HR: 0.82; 95% CI: 0.71 to 0.96; *p* = 0.01). In the VOYAGER study, the primary efficacy outcome (cardiovascular death, acute limb ischemia, major amputation, myocardial infarction, or stroke) occurred in 17.3% of the rivaroxaban-plus-aspirin group compared with 19.9% of the placebo/aspirin-alone group (*p* = 0.0085). The results of these studies have been considered in the most recent guidelines, which recommend this DPI (aspirin plus a low dose of rivaroxaban, 2.5 mg × 2 die) approach in patients with polyvascular disease, i.e., prior myocardial infarction (MI) or chronic coronary artery disease, to reduce MACE, and in patients with previous revascularization, who are at the highest risk of new vascular limb events, to reduce MALE [6,7,8]. These recent scientific conclusions have shown that more powerful antithrombotic approaches reduce MACE and MALE but increase bleeding in patients with PAD.

## 2. The Risk of Bleeding in Patients with PAD

Patients included in the COMPASS study with chronic coronary artery and chronic PAD who were treated with DPI compared to those treated with aspirin alone showed a 1.7-fold increased risk of major and minor bleeding. The bleeding events were most evident in the stomach, duodenum, colon, and rectum, and they were present in one-third of the cases from an unidentified source. Although no information was collected on how the bleeding events were detected, it is likely that most of the bleeding events of undefined origin derived from the small intestine, with an inflammatory or malformed vascular substrate that was not detected by normal endoscopic procedures. The highest risk of bleeding was observed in the first year, with no significant increase in the subsequent periods. The benefit in preventing MACE and MALE events, on the other hand, continued to increase over time, maintaining a considerable net clinical benefit [9]. It is likely that in the initial period of the most aggressive antithrombotic therapies, such as DPI, underlying pathologies that were not previously known could be revealed. Antithrombotic therapy and revascularization procedures entail an increased hemorrhagic risk that is also linked to more vigorous antithrombotic therapies. As previously mentioned, patients with severe PAD benefit from revascularization to improve symptoms, especially those related to chronic critical limb ischemia (rest pain and cutaneous ischemic lesions with tissue loss) or acute ischemia. The correlation between revascularization procedures and hemorrhagic complications is another point that is not well-defined and is still debated in PAD since most of the already-known evidence on bleeding after revascularization procedures is derived from studies on ischemic heart disease. A recent post hoc analysis from the EUCLID trial was conducted to evaluate the incidence of post-procedural bleeding. EUCLID was a randomized controlled clinical trial evaluating 13,855 patients with symptomatic PAD that assessed the efficacy and safety of ticagrelor compared to clopidogrel for the prevention of MACE. Of these patients, 2661 underwent a total of 3062 coronary (770) and peripheral (1905) revascularizations and amputations (387). The patients were randomized for treatment with clopidogrel or ticagrelor. Major and minor bleeding events were considered, as were the characteristics related to timing and severity. The incidence of major or minor bleeding after lower limb revascularization procedures was 3.3% at 7 days, comparable with that of coronary revascularization procedures and lower limb amputations, and most of the hemorrhagic events occurred at 7 days after the procedures. The severity of bleeding occurrences, defined according to ISTH criteria, at 30 days after the peripheral revascularization procedures was also similar to those of coronary and lower limb revascularization and lower limb amputation. Bleeding events were more frequent, although not statistically significant, in the ticagrelor group [10].

The literature suggests that female patients are more prone to developing postprocedural bleeding, and female sex is an independent predictor of bleeding complications after procedures of revascularization, especially after endovascular revascularization treatments, and this bleeding risk persists over time. The role of sex should be considered in the choice of antithrombotic regimen after endovascular treatment and evaluated in patient management after peripheral revascularization procedures to minimize bleeding. Recent data from a retrospective cohort study of nearly 32,000 patients undergoing endovascular treatment of the lower extremities showed a higher number of bleeding complications, with a risk difference (RD) of 0.028 and *p* < 0.01, with blood transfusions (RD 0.029 and *p* < 0.01) in females versus males at one-year follow-up. The utilization of antithrombotic and, particularly, anticoagulant therapy significantly impacted the patient outcomes. Antiplatelets were used in 63% of the cases, and among the anticoagulants used in 26% of cases, warfarin was the most common. A minority of patients were treated with double antiplatelet and anticoagulation, and only a very small proportion did not receive anti-thrombotics after surgery. The groups divided by gender were perfectly comparable for treatments. The authors speculated that different anticoagulant drug metabolisms in females may have contributed to the increase in bleeding complications, although the mechanism for this was unclear [11].

The risk of bleeding in patients with PAD who are candidates for revascularization procedures was considered in a position paper by the European Society of Vascular Medicine (ESVM). The authors evaluated the management of symptomatic PAD in patients who were possible candidates for endovascular limb procedures. Regarding the pre-interventional antithrombotic treatment, if patients were on DPI before the procedure, the low dosage rivaroxaban was interrupted peri-interventionally and aspirin was maintained. The dual antiplatelet therapy (DAPT) was continued, even with an increasing bleeding risk, if there is a cardiological indication and, consequently, an increased risk of MACE. In the post-procedural medication strategy, DPI was adopted to reduce MALE, provided the risk of bleeding was low, which was aligned with the ESC recommendations [7]. The authors concluded the study by proposing a number of open issues that need to be developed, including risk scores to predict thrombotic complications and restenosis, as well as scores to assess hemorrhagic risk [12]. 

## 3. A Personalized Approach between Thrombotic and Hemorrhagic Risk

**Figure 2:**
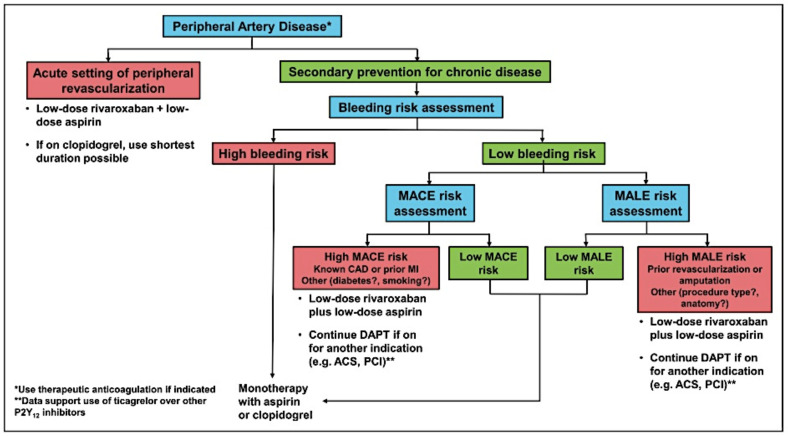
Algorithm to guide the choice of antithrombotic therapy in PAD, according to the clinical setting and the bleeding and ischemic risk (reprinted with permission from Ref. [3]. Copyright 14 December 2022. Wolters Kluwer Health, Inc.)

The identification of patients with PAD who are at higher risk of MACE and MALE and who achieve greater absolute advantages to compensate for the risk of bleeding is of clinical interest, as is the stratification of bleeding risk, which deals with the heterogeneity of risk according to clinical characteristics. The personalized approach to a patient with PAD is complex and must take into account the ischemic profile, with the risk of MALE in the acute phase setting and the risk of MACE in the chronic phase setting. However, this approach cannot disregard the assessment of bleeding risk. In extremely frail patients with PAD, the factors determining a high ischemic risk (i.e., critical limb ischemia, chronic renal disease, and diabetes) are the same factors that determine a high bleeding risk [3,13]. Hess and Bonaca have proposed an algorithm (Figure 2) to assess, in both the acute and chronic settings, the best antithrombotic therapy considering both the thrombotic and hemorrhagic risk. The choice of antithrombotic treatment for secondary prevention in chronic PAD is not well-defined. A preliminary evaluation of patients who are candidates for more aggressive antithrombotic therapy in secondary prevention for PAD should be comprehensive, and it should include an assessment of their bleeding risk. Patients at high risk of bleeding should receive a monotherapy with aspirin or clopidogrel. Patients at low risk of bleeding should be assessed for their ischemic risk. In the chronic setting of PAD, the risk of MACE prevails. Several significant risk factors for MACE are concomitant coronary artery disease (CAD) (especially in patients with previous myocardial infarction), diabetes, chronic renal disease, and heart failure. In these patients with PAD and increased risk for MACE, the use of low-dosage rivaroxaban plus low-dose aspirin is recommended [6]. Nevertheless, patients already undergoing DAPT for recent acute coronary syndrome or coronary percutaneous angioplasty are advised to continue with this therapy to prevent MACE and MALE. Again, in the context of chronic PAD, the most important risk factor for MALE is having had a previous peripheral revascularization or amputation, especially if it was performed for critical limb ischemia or acute limb ischemia. Treatment with low-dosage rivaroxaban plus low-dose aspirin is recommended for PAD patients with a high risk for MALE [6]. Patients already undergoing DAPT for recent acute coronary syndrome or coronary percutaneous angioplasty are also advised to continue with this therapy [3,13].

Paradoxically, bleeding significantly expands the risk of consequent ischemic events in patients with CAD, cerebrovascular disease (CVD), and antithrombotic treatment. Van Huttom and colleagues [14] evaluated the impact of bleeding in patients receiving oral anticoagulation or aspirin after peripheral bypass surgery in a considerable multicenter randomized trial (the Dutch Bypass and Oral Anticoagulants or Aspirin (BOA) Study). The patients who developed major bleeding were older, suffered from critical ischemia, diabetes, and hypertension, and were more frequently treated with antithrombotic therapies, including anticoagulants. The authors provided the first insight into the independent adverse effect of bleeding on subsequent ischemic events in a large trial representing the general population of patients with PAD who are treated with oral anticoagulants or aspirin after peripheral bypass surgery. These observations agree with previous evidence in studies of patients suffering from CAD or CVD, and they invite the use of the best antithrombotic therapy and risk control to successfully decrease ischemic events in the interim, decreasing the risk of bleeding [14]. There are a few hypotheses to explain the association between bleeding and subsequent thrombotic risk. One hypothesis identifies discontinuation of antithrombotic therapy as the greatest subsequent thrombotic risk. A second hypothesis suggests that patients who have experienced a bleeding complication during antithrombotic therapy are more frail patients with critical limb ischemia and renal failure. A third consideration concerns hypotension, anemia, and blood transfusions following bleeding. In particular, anemia is associated with death, repeated revascularizations, or myocardial infarctions [14].

## 4. The Importance of Clinical Risk Scores

Most randomized controlled trials (RCTs) comparing different antithrombotic strategies have not considered patients with elevated hemorrhagic risk. Therefore, it becomes difficult to determine the optimal therapy for subjects who do not present a co-existing indication for oral anticoagulation or enhanced antithrombotic treatment. In patients with PAD, unfortunately, there is a scarcity of knowledge about the safety of the anti-thrombotic approach. Doctors must balance the risk profile of patients with PAD to adjust the personal necessity for an increased anti-thrombotic therapy that is not in favor of the incremental risk of life-threatening hemorrhages. For this reason, clinical risk scores could help to adapt the therapeutic approach to increase ischemic protection and reduce hemorrhagic risk in a specific individual. Regrettably, several factors are associated with both hemorrhagic and thrombotic problems, and so predicting advantages and disadvantages becomes a difficult and complicated task. A large number of risk scores integrate demographic elements, previous and contextual illnesses, and procedural evaluations to lead physicians in choosing the best antithrombotic therapy. Most obtainable risk scores were originally developed and validated for coronary artery disease, with an undefined applicability to PAD cohorts. 

The HAS-BLED score was created by the Euro Heart Survey to evaluate the one-year risk of major bleeding in individuals with atrial fibrillation [15]. The HEMORR2HAGES score utilizes Medicare beneficiaries with atrial fibrillation [16]. 

In recent years, the ARC-HBR consensus document, the primary realistic method used to develop a coherent description of elevated hemorrhagic risk in clinical trials, has been particularly referred only for patients undergoing percutaneous coronary interventions [17].

## 5. Bleeding Risk Assessment in Patient with PAD

We have reviewed a number of reports aimed at assessing bleeding risk in patients with PAD that could help us define the best antithrombotic therapy for PAD patients by assessing not only thrombotic risk, but also bleeding risk, which is, paradoxically, often associated with increased thrombotic risk and a poorer prognosis [18,19]. 

Ducrocq and colleagues developed a risk score using the REACH Registry to quantify bleeding risk in a cohort of 68,236 outpatients with or at risk of atherothrombosis [20]. The Reduction of Atherothrombosis for Continued Health (REACH) Registry, a prospective registry of patients aged at least 45 years, recruited patients with known CVD, CAD, or PAD, or patients with at least three atherosclerosis risk factors. This score was validated externally using the CHARISMA study, which was aimed at evaluating the possible benefits of dual antiplatelet treatment in patients with previous myocardial infarction, ischemic stroke, or symptomatic PAD. The definitive bleeding risk score sheet included one demographic factor (age, 2–6 points); two predictors associated with medical history (PAD, 1 point; CHF (chronic heart failure), 2 points); four comorbidities or lifestyle features (diabetes, 1; hypercholesterolemia, 1; hypertension, 2; smoking, 1–2); and two medication regimens (antiplatelets, 1–4; oral anticoagulants, 4). The highest score possible is 23, though the maximum recorded score was 21. This score very closely approximated the complete nine-factor regression model, with a correlation of 0.993 between the predicted probabilities from the regression and the score.

**Figure 3:**
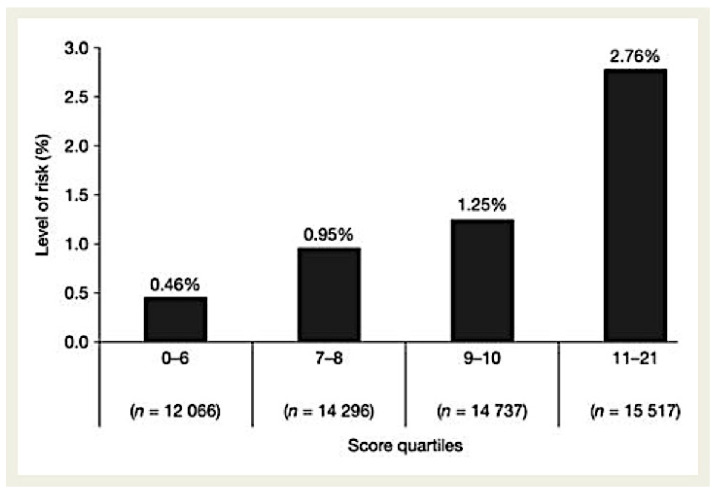
Risk stratification using the REACH score (percentages indicate the 2-year risk of serious bleeding) (reprinted with permission from Ref. [20]. Copyright 24 February 2010, Oxford University Press).

Risk stratification using this score was quite effective in classifying the risk level of patients, with a more than six-fold increase in risk between the highest and lowest quartiles. As shown in Figure 3, there was a 2-year incidence of 0.46% in patients with a score of ≤6 vs. 2.76% in patients with a score of ≥11.

This risk score prognosticates important clinical hemorrhages in stable outpatients with, or at risk of, atherothrombotic disease, and it exemplifies an important approach for dealing with outpatients suffering from PAD. However, some of the features and risk profiles of hospitalized cohorts who undergo revascularization may diverge considerably. In addition, the score fails to consider the clinical severity of PAD. 

In Table 1 we reported the principal studies evaluating bleeding in patients with PAD. 

As a setting for intensifying antithrombotic strategies to prevent MACE and MALE, Baumann and colleagues evaluated bleeding risk using the HAS-BLED score in 115 consecutive patients with symptomatic PAD undergoing endovascular revascularization. Logistic regression analysis identified aortoiliac or femoropopliteal segment involvement, CKD, and the Rutherford category 5/6 as independent risk factors related to a HAS-BLED score of ≥3. The HAS-BLED scale would represent a great clinical instrument for evaluating the risk–benefit ratio of antithrombotic therapy in PAD patients, especially for those with critical limb ischemia. However, the HAS-BLED score requires prospective validation for its use in PAD patients [21].

A retrospective, observational cohort study collected data from The Health Improvement Network (THIN) database in the United Kingdom [22]. THIN is an electronic medical research database that includes fully anonymized data on more than 3.7 million patients currently enrolled with participating primary care practices in the United Kingdom. Patients with data in THIN who were aged 50 to 89 years at any time between 1 January 2000 and 31 December 2010 were incorporated in the study. In this real-world clinical practice context, the authors evaluated potential predictors of intra-cranial bleeding (ICB) and gastrointestinal bleeding (GIB) in patients with symptomatic PAD. The results revealed differences in risk factor models for the two bleeding outcomes. For ICB, the predictors of increased risk included previous ICB, dementia, low BMI, osteoporosis, and falls in the previous year. Osteoporosis is more common among women than men, and older women with low bone mineral density are at higher risk of atherosclerotic disease incorporating PAD; this is another situation in which thrombotic risk and hemorrhagic risk may coexist. Polytherapy, statin use, and the utilization of anti-hypertensive treatment were predictors of a reduced risk of ICB. Anti-platelet therapy and the use of NSAIDs were major predictors for GIB. The authors advocated the development of a bleeding risk score which could improve treatment strategies for patients with symptomatic PAD [22].

De Vries and collaborators proposed the evaluation of a complete individual lifetime advantage and the damage of low-dosage rivaroxaban (2.5 mg bid) treatment in association with acetylsalicylic acid for specific patients with stable cardiovascular disease. Their benefit–harm analysis, utilizing subjects registered to the COMPASS and SMART trials, assessed probable life-years without myocardial infarction or stroke and probable life-years gained due to missed major hemorrhages. The investigators observed that the lifetime treatment outcomes of low-dosage rivaroxaban treatment associated with acetylsalicylic acid in specific patients with stable cardiovascular disease could be considered utilizing the individuals’ features, allowing for lifetime acquisitions and dangers to be weighed for individual patients. Nevertheless, the c-indexes for the lifetime scores persisted as moderate for a predictive design used for individualizing long term secondary prevention. Moreover, the utility of this instrument could be decreased by the inclusion of data from COMPASS, an interventional trial, and from SMART, an observational study. Additionally, the utilization of information from SMART patients recruited in 1996, when the standard of treatments was completely dissimilar, is questionable [23].

A tool for assessing bleeding risk has also been provided also by the Academic Research Consortium for High Bleeding Risk (ARC-HBR) [17]. Patients are considered to be at high bleeding risk if at least one major or two minor criteria are met. The major criteria are anticipated use of long-term oral anticoagulation; severe or end-stage CKD (eGFR levels of <30 mL/min); hemoglobin levels of <11 g/dL; spontaneous bleeding requiring hospitalization or transfusion in the past 6 months (or at any time, if recurrent); moderate or severe baseline thrombocytopenia (platelet count of <100 × 10^9^/L); chronic bleeding diathesis; liver cirrhosis with portal hypertension; active malignancy (excluding nonmelanoma skin cancer) within the past 12 months; previous spontaneous ICH (at any time); previous traumatic ICH within the past 12 months; presence of a brain arteriovenous malformation; moderate or severe ischemic stroke within the past 6 months; non-deferrable major surgery on dual antiplatelet therapy; and recent major surgery or major trauma within 30 d before receiving percutaneous coronary interventions (PCI). The minor criteria are age of ≥75 years; moderate CKD (eGFR levels of 30–59 mL/min); hemoglobin levels of 11–12.9 g/dL for men and 11–11.9 g/dL for women; spontaneous bleeding requiring hospitalization or transfusion within the past 12 months that does not meet the major criterion; long-term use of oral NSAIDs or steroids; and any ischemic stroke at any time that does not meet the major criterion. This model is simple, practical, and accurate, but it has been proposed to provide a structure for estimating alternative medications for patients undergoing PCI with at elevated hemorrhagic risk.

**Figure 4:**
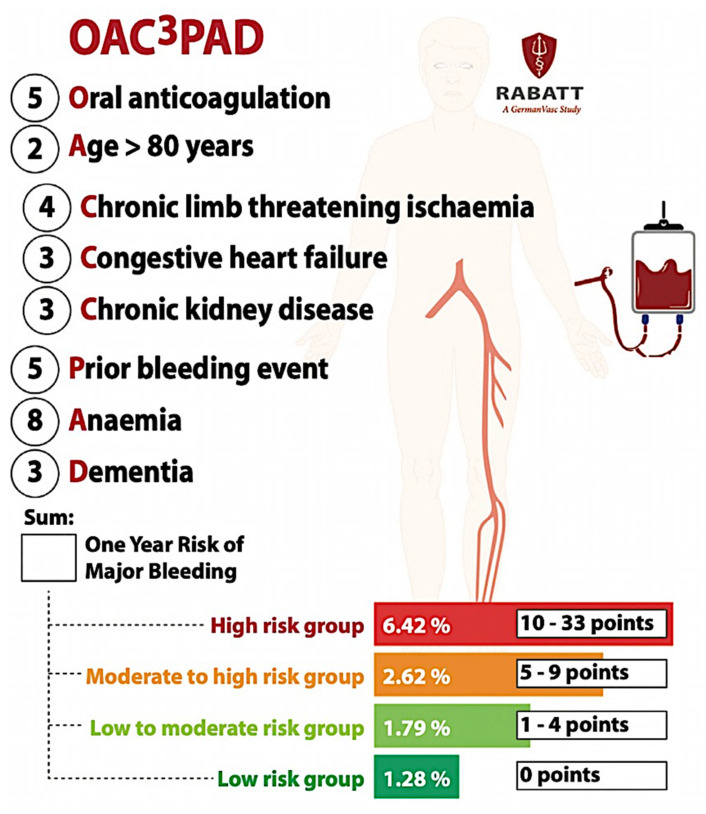
OAC^3^-PAD bleeding risk score considering the following four risk categories: low; low-to-moderate; moderate-to-high; and high. The numbers before the risk factors are the point scores to be summarized for the presence of this risk factor (reprinted from [24]).

Recently, a realistic risk score including eight independent variables was created, and its individual prediction scores can support the risk and advantage assessment of an intensified antithrombotic approach [24]. Unselected retrospective information from the second largest insurance fund in Germany, BARMER, was utilized for identifying patients with a first hospitalization for PAD, registered between 1 January 2010 and 31 December 2018. Inside of a different training group, final predictors were identified using a penalized Cox regression, with one-year major bleeding necessitating hospitalization as outcome. The risk score was internally corroborated. Four distinctive risk categories were defined. The OAC-PAD bleeding score included the following eight unrelated predictors: oral anticoagulant therapy (5 points), age above 80 (2 points), chronic limb threatening ischemia (4 points), congestive heart failure (3 points), severe CKD (3 points), prior bleeding event (5 points), anemia (8 points), and dementia (3 points). The definitive four risk categories were: (1) a low-risk group with 0 points, (2) a low-to-moderate-risk group with 1 to 4 points, (3) a moderate-to-high-risk group with 5 to 9 points, and (4) a high-risk group with 10 to 33 points, associated with approximated risk levels of 1.28%, 1.79%, 2.62%, and 6.42%, respectively (Figure 4) [24]. These personal prediction scores can help to evaluate the risks and advantages of increased antithrombotic therapies in patients with PAD, in addition to taking into account the severity of the diseases to which several more scores refer. It is usable for patients undergoing both endovascular and open surgical revascularizations.

## 6. Conclusions

Bleeding risk in patients with cardiovascular disease and PAD is mutable. This risk continuum is improperly estimated as a ‘on - off’ evaluation at the beginning of treatment to define situations many years later and considers probable alterations by ageing, occasional risk factors, or modifying associated pathologies. Therefore, with the introduction of associated anticoagulant and antiplatelet treatments, the hemorrhagic risks of patients should be reconsidered frequently. Consequently, the correct application of a risk score can be used to determine variable bleeding risk factors that could be revised, as well as to identify patients with elevated risks for additional regular reexamination and control. Clinical risk scores absolutely do not represent the total reality and inter-score variability that must be taken into account. Moreover, several risk scores are basic, and they become facilitators used to improve everyday decision making. Many risk scores based on points vary according to the configuration of the study, population variety, ethnic type, etc. In addition, several of the risk factor elements in a specific score are unlikely to sustain the same risk weight. The best approach continues to be devising an uncomplicated, functional, validated, and precise score that can be adjusted to different clinical contexts and populations, and that considers the mutable composition of clinical risk.

## Figures and Tables

**Table 1:** A summary of the articles evaluating bleeding in patients with PAD.

	Therapy	Type of Study
Baumann 2016 [21]	Antiplatelet drugs or anticoagulants	Prospective study on symptomatic PAD patients undergoing endovascular revascularization
Cea Soriano 2018 [22]	Antiplatelet drugs or anticoagulants	Retrospective study on PAD patients
De Vries 2019 [23]	Low dose rivaroxaban + aspirin	COMPASS study (interventional) and SMART Study (observational)
Behrendt 2022 [24]	Antiplatelet drugs or anticoagulants	Retrospective study on patients with a first hospitalization for PAD

## Data Availability

The data are available upon request from the corresponding author.

## References

[1] Fowkes F.G., Rudan D., Rudan I., Aboyans V., Denenberg J.O., McDermott M.M., Norman P.E., Sampson U.K., Williams L.J., Mensah G.A. (2013). Comparison of global estimates of prevalence and risk factors for peripheral artery disease in 2000 and 2010: A systematic review and analysis. Lancet.

[2] Hess C.N., Wang T.Y., Weleski Fu J., Gundrum J., Allen LaPointe N.M., Rogers R.K., Hiatt W.R. (2020). Long-term outcomes and associations with major adverse limb events after peripheral artery revascularization. J. Am. Coll. Cardiol..

[3] Hess C.N., Bonaca M.P. (2020). Contemporary Review of Antithrombotic Therapy in Peripheral Artery Disease. Circ. Cardiovasc. Interv..

[4] Eikelboom J.W., Connolly S.J., Bosch J., Dagenais G.R., Hart R.G., Shestakovska O., Diaz R., Alings M., Lonn E.M., Anand S.S. (2017). Rivaroxaban with or without Aspirin in Stable Cardiovascular Disease. N. Engl. J. Med..

[5] Bonaca M.P., Bauersachs R.M., Anand S.S., Debus E.S., Nehler M.R., Patel M.R., Fanelli F., Capell W.H., Diao L., Jaeger N. (2020). Rivaroxaban in Peripheral Artery Disease after Revascularization. N. Engl. J. Med..

[6] Frank U., Nikol S., Belch J., Boc V., Brodmann M., Carpentier P.H., Chraim A., Canning C., Dimakakos E., Gottsäter A. (2019). ESVM Guideline on peripheral arterial disease. Vasa.

[7] Aboyans V., Bauersachs R., Mazzolai L., Brodmann M., Palomares J.F.R., Debus S., Collet J.P., Drexel H., Espinola-Klein C., Lewis B.S. (2021). Antithrombotic therapies in aortic and peripheral arterial diseases in 2021: A consensus document from the ESC working group on aorta and peripheral vascular diseases, the ESC working group on thrombosis, and the ESC working group on cardiovascular pharmacotherapy. Eur. Heart J..

[8] Belch J.J.F., Brodmann M., Baumgartner I., Binder C.J., Casula M., Heiss C., Kahan T., Parini P., Poredos P., Catapano A.L. (2021). Lipid-lowering and anti-thrombotic therapy in patients with peripheral arterial disease. Vasa.

[9] Eikelboom J.W., Bosch J.J., Connolly S.J., Shestakovska O., Dagenais G.R., Hart R.G., Leong D.P., O’Donnell M., Fox K.A.A., Bhatt D.L. (2019). Major Bleeding in Patients with Coronary or Peripheral Artery Disease Treated with Rivaroxaban Plus Aspirin. J. Am. Coll. Cardiol..

[10] Kansal A., Huang Z., Rockhold F.W., Baumgartner I., Berger J.S., Blomster J.I., Fowkes F.G., Katona B., Mahaffey K.W., Norgren L. (2019). Impact of Procedural Bleeding in Peripheral Artery Disease: An Analysis From EUCLID Trial. Circ. Cardiovasc. Interv..

[11] Marulanda K., Duchesneau E., Patel S., Browder S.E., Caruso D.M., Agala C.B., Kindell D.G., Curcio J., Kibbe M.R., McGinigle K. (2022). Increased long-term bleeding complications in females undergoing endovascular revascularization for peripheral arterial disease. J. Vasc. Surg..

[12] Heiss C., Olinic D.M., Belch J.J.F., Brodmann M., Mazzolai L., Stanek A., Madaric J., Krentz A., Schlager O., Lichtenberg M. (2022). European Society of Vascular Medicine. Management of chronic peripheral artery disease patients with indication for endovascular revascularization. Vasa.

[13] Bonaca M.P., Hamburg N.M., Creager M.A. (2021). Contemporary Medical Management of Peripheral Artery Disease. Circ. Res..

[14] Van Hattum E.S., Algra A., Lawson J.A., Eikelboom B.C., Moll F.L., Tangelder M.J. (2009). Bleeding increases the risk of ischemic events in patients with peripheral arterial disease. Circulation.

[15] Pisters R., Lane D.A., Nieuwlaat R., De Vos C.B., Crijns H.J., Lip G.Y. (2010). A novel user-friendly score (HAS-BLED) to assess 1-year risk of major bleeding in patients with atrial fibrillation: The Euro Heart Survey. Chest.

[16] Gage B.F., Yan Y., Milligan P.E., Waterman A.D., Culverhouse R., Rich M.W., Radford M.J. (2006). Clinical classification schemes for predicting hemorrhage: Results from the National Registry of Atrial Fibrillation (NRAF). Am. Heart J..

[17] Urban P., Mehran R., Colleran R., Angiolillo D.J., Byrne R.A., Capodanno D., Cuisset T., Cutlip D., Eerdmans P., Eikelboom J. (2019). Defining high bleeding risk in patients undergoing percutaneous coronary intervention: A consensus document from the Academic Research Consortium for High Bleeding Risk. Eur. Heart J..

[18] Toyoda K., Yasaka M., Iwade K., Nagata K., Koretsune Y., Sakamoto T., Uchiyama S., Gotoh J., Nagao T., Yamamoto M. (2008). Bleeding with Antithrombotic Therapy (BAT) Study Group. Dual antithrombotic therapy increases severe bleeding events in patients with stroke and cardiovascular disease: A prospective, multicenter, observational study. Stroke.

[19] Eikelboom J.W., Mehta S.R., Anand S.S., Xie C., Fox K.A., Yusuf S. (2006). Adverse impact of bleeding on prognosis in patients with acute coronary syndromes. Circulation.

[20] Ducrocq G., Wallace J.S., Baron G., Ravaud P., Alberts M.J., Wilson P.W., Ohman E.M., Brennan D.M., D’Agostino R.B., Bhatt D.L. (2010). Risk score to predict serious bleeding in stable outpatients with or at risk of atherothrombosis. Eur. Heart J..

[21] Baumann F., Husmann M., Benenati J.F., Katzen B.T., Del Conde I. (2016). Bleeding Risk Profile in Patients with Symptomatic Peripheral Artery Disease. J. Endovasc. Ther..

[22] Cea Soriano L., Fowkes F.G.R., Allum A.M., Johansson S., García Rodriguez L.A. (2018). Predictors of Bleeding in Patients with Symptomatic Peripheral Artery Disease: A Cohort Study Using The Health Improvement Network in the United Kingdom. Thromb. Haemost..

[23] De Vries T.I., Eikelboom J.W., Bosch J., Westerink J., Dorresteijn J.A.N., Alings M., Dyal L., Berkowitz S.D., Van der Graaf Y., Fox K.A.A. (2019). Estimating individual lifetime benefit and bleeding risk of adding rivaroxaban to aspirin for patients with stable cardiovascular disease: Results from the COMPASS trial. Eur. Heart J..

[24] Behrendt C.A., Kreutzburg T., Nordanstig J., Twine C.P., Marschall U., Kakkos S., Aboyans V., Peters F. (2022). The OAC^3^-PAD Risk Score Predicts Major Bleeding Events one Year after Hospitalisation for Peripheral Artery Disease. Eur. J. Vasc. Endovasc. Surg..

